# Bimetallic Coordination Polymers: Synthesis and Applications in Biosensing and Biomedicine

**DOI:** 10.3390/bios14030117

**Published:** 2024-02-22

**Authors:** Yanping Sun, Jianxin Ma, Faisal Ahmad, Yelan Xiao, Jingyang Guan, Tong Shu, Xueji Zhang

**Affiliations:** 1 Beijing Key Laboratory for Bioengineering and Sensing Technology, School of Chemistry and Biological Engineering, University of Science and Technology Beijing, Beijing 100083, China; 2 Shenzhen Key Laboratory for Nano-Biosensing Technology, Research Center for Biosensor and Nanotheranostic, Guangdong Key Laboratory of Biomedical Measurements and Ultrasound Imaging, School of Biomedical Engineering, Shenzhen University Medical School, Shenzhen University, Shenzhen 518060, China; faisal@email.szu.edu.cn (F.A.);; 3 Department of Molecular and Cellular Pharmacology, School of Pharmaceutical Sciences, Peking University, Beijing 100191, China

**Keywords:** bimetallic, coordination polymers, synergistic effects, biosensing, biomedicine

## Abstract

Bimetallic coordination polymers (CPs) have two different metal ions as connecting nodes in their polymer structure. The synthesis methods of bimetallic CPs are mainly categorized into the one-pot method and post-synthesis modifications according to various needs. Compared with monometallic CPs, bimetallic CPs have synergistic effects and excellent properties, such as higher gas adsorption rate, more efficient catalytic properties, stronger luminescent properties, and more stable loading platforms, which have been widely applied in the fields of gas adsorption, catalysis, energy storage as well as conversion, and biosensing. In recent years, the study of bimetallic CPs synergized with cancer drugs and functional nanomaterials for the therapy of cancer has increasingly attracted the attention of scientists. This review presents the research progress of bimetallic CPs in biosensing and biomedicine in the last five years and provides a perspective for their future development.

## 1. Introduction

Coordination polymers (CPs) or coordination networks are inorganic or organometallic polymer structures with infinite structures formed through the self-assembly of transition metals and organic ligands [1,2,3]. CPs exhibit unique properties by combining the characteristics of both polymers and coordination compounds [4]. Since the concept of CPs was introduced in 1964, a large number of CPs with different structures and compositions have been designed and synthesized using various metal ions and organic ligands under different reaction conditions [5,6,7]. Some of them are able to form crystal morphology and X-ray crystallography can then be used to resolve their structural composition at the atomic level [8]. Among them, metal-organic frameworks (MOFs) are currently one of the most studied classes due to their rigid [9], ordered, and highly porous structures, which have been found widely potentials in gas storage and separation [10,11,12], catalysis [13,14,15], sensing [16,17], and biomedicine [18].

Bimetallic CPs comprised of second metal ions in the coordination node have abundant structures and compositions, ensuring their more versatile properties and application in comparison with monometallic CPs. The presence of two metal ions in bimetallic CPs produces synergistic and cooperative effects on their functionality, and the ratio of the metal ions can be adjusted, offering the possibility of controllable synthesis of bimetallic CPs with specific physicochemical properties [19,20]. For example, bimetallic d-f CPs were constructed using d-block metal chromophores as sensitizers to achieve luminescence through effective d → f energy transfer, which effectively overcame the problem of low f → f conversion efficiency [21]. In the field of catalysis, for example, the NiFeCP/NF (NF = nickel foam, terephthalate as the ligand) electrode exhibited excellent oxygen evolution reaction (OER) catalytic activity and was a promising catalyst for oxygen-absorbing materials [22]. Magnetic bistable materials with hysteretic properties, which consisted of parallel cyanide bridge [Fe^II^-W^v^] coordination chains linked together by rigid double (imidazole) benzene ligands, were comparable to basic binary units and were expected to be used in switching and memory devices [23]. In the field of environment, compared with the monometallic zinc CPs, the bimetallic CPs contain [Zn_2_M_2_O]^6+^ (M = Co or Ni) bimetallic cluster nuclei and more open metal sites (OMS) showed distinct isosteric heats of adsorption and surface area, owing to their open Lewis acidic sites of solvent-free state. Moreover, bimetallic CPs can also remove oil spills from water surfaces in powder and particle form with a clearance rate of up to 385 wt% (clearance rate = mass of adsorbed oil spill/mass of materials), providing a roadmap for the design and manufacture of novel superhydrophobic porous composite materials in combination with OMS to offer better water and thermal stability [24]. These works have been well reviewed and discussed for their potential in gas storage [25] and catalysis [26]

**Figure 1:**
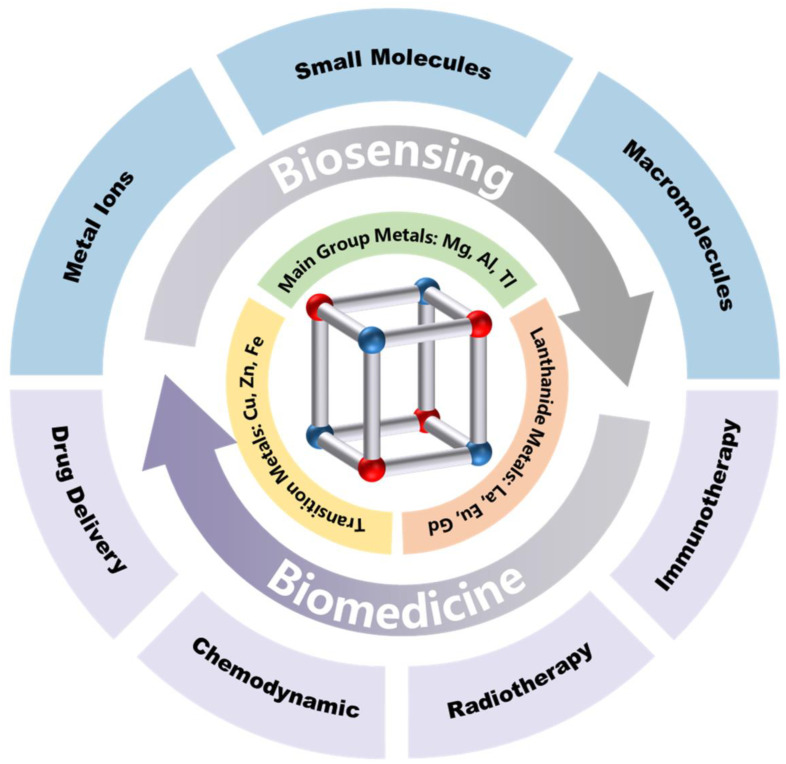
Schematic diagram of the constituent elements of bimetallic CPs and their applications in biosensing and biomedicine.

For another aspect, bimetallic CPs with exotic structure of porous morphology and regular topologies have intriguing optical and electronic properties, favoring their ability in biosensing and biomedicine [27,28]. However, there is still a lack of comprehensive discussion dedicated to the fields of bio-related applications. This paper describes recent advances in bimetallic CPs for biosensing and biomedical applications with the aim of filling this gap (Figure 1). Synthesis strategies of bimetallic CPs including the one-pot method and post-synthesis modifications are first classified and discussed. The applications of bimetallic CPs in sensing and drug delivery fields such as luminescent probes, electrochemical sensing, enzyme mimicry, drugs, and immunotherapy for cancer are then successively presented. Finally, this review offers an outlook for the possible development of bimetallic CPs in biological applications.

## 2. The Synthetic Strategies for Bimetallic CPs

CPs contain two core components, connectors and linkers, which are defined as the original reagents that build the main framework of the CPs. Their important characteristics are the number and orientation of their binding sites (coordination number and coordination geometry). Transition metal ions are commonly employed as multifunctional connectors in structures of CPs and the coordination number changes depending on the type of metal and its oxidation state. Organic molecules and anions often act as linkers providing abundant attachment sites and tuning the strength and orientation of the binding [1]. In addition, a wide variety of CP materials have been successfully synthesized by introducing different synthesis conditions from coordination chemistry and zeolite chemistry, including room temperature (RT), conventional electric (CE), microwave heating (MW), electrochemical (EC), mechanochemistry (MC), and ultrasonic (US) methods [29].

This section focuses on two strategies for the synthesis of bimetallic CPs: one-pot methods and post-synthesis modifications. One-pot synthesis refers to the process of the second ion added before the formation of polymer structure. On the contrary, post-synthesis modifications are the methods of adding a second ion after the polymer structure has been constructed. The development of instrumental analysis has provided technical support for characterizing the composition and structure of bimetallic CPs. X-ray diffraction (XRD) can be used to identify the crystalline phase of bimetallic CPs [30]. Atomic absorption spectroscopy (AAS) and energy dispersive X-ray spectroscopy (EDX) can be coupled to calculate the concentration and distribution of the two metals in the CPs [31]. X-ray photoelectron spectroscopy (XPS) and X-ray absorption fine structure analysis (XAFS) can determine the nodal position of each metal in the bimetallic CPs [32,33]. The specific surface area, pore volume, and pore size distribution of the bimetallic CPs can be determined from the N_2_-sorption desorption isotherm [34]. The combination of different techniques to accurately characterize the synthetic bimetallic CPs exploits the potential for applications in various fields [35,36,37].

### 2.1. One-Pot Methods

**Figure 2:**
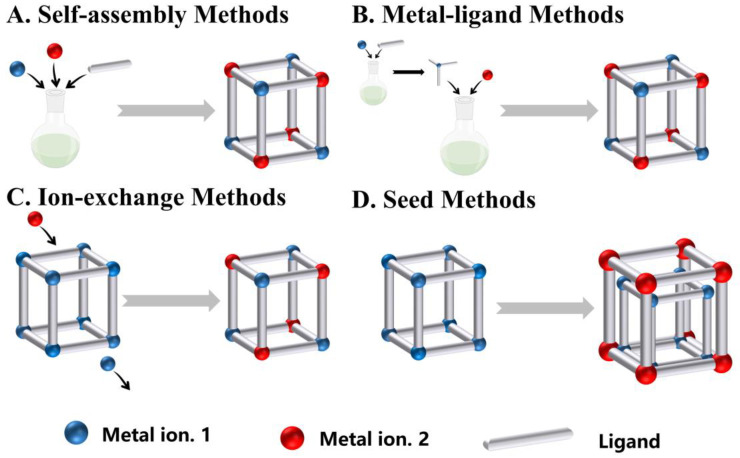
One-pot methods for bimetallic CP synthesis. (**A**) self-assembly methods, (**B**) metal-ligand methods and post-synthesis modifications, (**C**) ion-exchange methods, (**D**) seed methods.

One-pot methods involve the addition of a second ion before the polymer structure has been constructed, which encompasses self-assembly methods of two metal ions with the ligand and metal-ligand methods with the metal-ligand as the site (Figure 2). The simplicity and rapidity of this method have led to widespread applications in the synthesis of bimetallic CP materials on a large scale.

#### 2.1.1. Self-Assembly Methods

Conventionally, monometallic CPs have been self-assembled by mixing organic ligands and metal salts in a one-pot manner [38,39]. Bimetallic CPs have similarly been synthesized in large quantities using self-assembly methods in past studies. The assembly process of bimetallic ions with organic ligands is usually disordered, and thus the synthesis of ordered self-assembly of bimetallic CPs requires specific reaction conditions such as solvothermal [40], ultrasonic [41], or microwave methods [42] to spontaneously change the system from disordered to ordered through the driving of internal forces. Meanwhile, parameters such as the type and concentration ratio of metal ions [43], as well as the pH of the reaction [44] need to be finely selected and controlled to achieve controlled doping.

Metal ion pairs with similar characteristics, such as ionic radius, charge, and Lewis acidity, are most often used to synthesize identically charged bimetallic CPs in a self-assembly manner due to the fact that two metal ions can form almost identical secondary-building units (SBUs), referring to the smallest repeating unit of CPs formed by one metal ion and multiple ligands. A large number of bimetallic CPs have been synthesized, for example Co^2+^-dopped ZIF-8(Zn^2+^) (ZIF stands for the zeolitic imidazolate framework) [45], Ce^3+^-dopped Tb-CPNs(Tb^3+^) (CPNs stand for coordination polymers nanoparticles) [46], Co^2+^-dopped Ni_3_HITP_2_(Ni^2+^) (HITP = 2,3,6,7,10,11-hexaiminotriphenylene) [47], Cu^2+^-dopped ZIF-67(Co^2+^) [48], Fe^3+^-dopped Cr-BTC(Cr^3+^) (BTC = 1,3,5-benzenetricarboxylic acid) [49], Hf^4+^-dopped Zr-MOF(Zr^4+^) [50] and Ce^4+^-dopped UiO-66(Zr^4+^) (UiO stands for University of Oslo, 1,4-benzenedicarboxylic acid as the ligand) [51]. The crystalline phases of the obtained bimetallic CPs are unchanged from those of monometallic CPs due to the almost identical coordination properties of these metal pairs. On the other hand, the self-assembly synthesis of ion pairs with different coordination abilities has also been widely reported such as Mg^2+^-Cr^3+^ MIL (MIL stands for Materials Institute Lavoisier, terephthalic acid as a ligand) [52], Ni^2+^-Gd^3+^ CPs [53], Fe^2+^-Gd^3+^ CPs [54], Fe^3+^-Ni^2+^ CPs [22], Na^+^-In^3+^ CPs [55], Cu^2+^-Zr^4+^ UiO-66 [56], Ag^+^-Zn^2+^ MOF [57] and Mn^2+^-Fe^3+^ MOF [58]. The addition of metal ions with different coordination abilities generates new SBUs leading to the changes in the crystalline phase, exhibiting different structures and properties. The simplicity and flexibility of the self-assembly methods have led to it being the most commonly used method for synthesizing bimetallic CPs.

#### 2.1.2. Metal-Ligand Methods

In order to control the synthesis of bimetallic CPs more efficiently, the use of designed SBUs is a promising regulatory approach. The metal-ligand methods refer to forming bimetallic CPs by reacting with target metal ions using metal complexes with donor sites instead of conventional organic ligands. Pre-synthesized metal complexes designed as SBUs can better control the structure and size of bimetallic CPs and endow them with superior properties.

Ferrocene (Fc) is a metal complex formed by the strong interaction of Fe (II) and cyclopentadienyl, which has good thermal stability and oxygen resistance thus facilitating the synthesis of various derivatives [59]. The synergistic effects of the excellent electrochemical properties of Fc and the structural properties of CPs have attracted great research interest in Fc-CP materials in the fields of electrochemistry and capacitors [60]. María et al. [61] first reported a 3D metal-organometallic network (MOMN) with a rhomboid-like network topology containing 1,1′-ferrocene dicarboxylate ligands. Two new distinct structures, [Zn(4,4′bipy)_2_(O_2_CFcCO_2_)_2_]·0.5H_2_O and [Cu(4,4′-bipy)_2_(O_2_CFcCO_2_)_2_] 0.5H_2_O, exhibiting a 3-fold interpenetrating structure with a topology according to point symbols for a net with loops, {6^6^}{6}2, have been successfully synthesized via hydrothermal reaction. The electrochemical properties of two Fc-CPs were evaluated by solution-state differential pulse voltammetry. Van Wyk and co-workers [62] have developed Fc@NU-1000 (NU-1000 = Zr-MOF) CPs using well-established pyrene-based MOF NU-1000 samples followed by modifying with ferrocene carboxylate via a SALI-based (SALI = solvent-assisted ligand incorporation) node functionalization technique. In dielectric-related charge transfer (CT) kinetic experiments, the CT products are immobilized in the porous NU-1000 framework, which avoid the interference of electrolyte counterions in changing the dielectric constant in conventional electrochemical experiments. It is shown that the process involves large reorganization energy, which requires polarization node-bound hydroxyl/water ligands, and the findings can provide an important reference for the future design of MOF-based electrocatalytic and photoelectrochemical systems.

Cyano (CN^−^)-based metal complexes are another widely used metal ligands in the synthesis of bimetallic CPs. For example, K_3_Fe(CN)_6_ was successively reacted with Cu^2+^ and Ni^2+^ to obtain core-shell nanoparticles of CuFe-PBA@NiFe-PBA (PBA = Prussian blue analogs) [63]. The [Nb(CN)_8_]^4−^ anion can bridge two [Mn(R-mpm)_2_]^2+^ units (mpm = α-methyl-2-pyridinemethanol) and a crystalline H_2_O molecule to form 2D cyano-bridged molecular ferromagnet {[Mn^II^(R-mpm)_2_]_2_[Nb^IV^(CN)_8_]}·4H_2_O [64]. In addition, [W^V^(CN)_8_]^3−^ building blocks and Fe^II^-based spin crossover (SCO) units can be linked to construct the cyanide-bridged alternating Fe^II^-W^V^ chain by the rigid diatopic bib ligands (bib = 1,4-bis(1H-imidazol-1-yl)benzene) to form a flexible framework{[W^V^(CN)_8_]-[(Fe^II^)(bib)_2_]-(bibH)}·2CH_3_OH [23]. 

Exceptionally, bimetallic coordination polymers with different valence states of the same element have been synthesized by the Metallo-ligand methods. Hou et al. reported that novel bimetallic CPs {[Cu^II^(SalImCy)](Cu^I^I)_2_·DMF}_n_ were fabricated by combining copper(II)-salen catalysts Cu^II^(SalImCy) (SalImCy = N,N’-bis-[(imidazol-4-yl)methylene]cyclohexane-1,2-diamine) with copper(I) iodide clusters via a direct solvothermal approach, which can be used as highly efficient multiphase multifunctional catalysts for the asymmetric synthesis of α-aminonitriles [65]. Suchithra et al. prepared bimetallic CPs of the [TAG][Fe^II^Fe^III^(ClCNAn)_3_]-(solvate) type (TAG = tris(amino)-guanidinium, ClCNAn^2−^ = chlorocyanoanilate dianionic ligand), which was the first report of the use of TAG in such CPs [66]. TAG cation has C3 symmetry and is able to form intermolecular hydrogen bonds with the chlorine atoms of the ligand and crystallize on the polar non-centrosymmetric space group P3. This paper followed up with an in-depth study of the magnetic and conductive properties of such Fe^II^-Fe^III^ CPs. The synthesis of bimetallic CPs with various functionalities requires the use of metal ligands with open metal sites and corresponding functionalities. Careful design and precise preparation of metal ligands are significant for this synthetic approach.

### 2.2. Post-Synthesis Modifications

Bimetallic CPs can be fabricated by synthesizing monometallic CPs followed by the addition of second ions, for example, ion exchange, template, and seed methods, whereas direct synthesis may not be possible. While the structure of CPs is preserved intact, the addition of new metal ions can bring new features and enhanced functions.

#### 2.2.1. Ion-Exchange Methods

Massive replacement of metal ions in CP systems by different metals with similar properties not only preserves the original structure but also provides additional functions [10]. The coordination number of SBUs, the valence of the metal ions, and the type of solvent have a significant impact on the rate and extent of the ion exchange [67]. 

Cu^2+^ is the most commonly used ion in ion exchange methods and tends to displace most of the other transition metal ions (Zn^2+^ and Mn^2+^) due to the energy gain by additional splitting of the d-orbitals by distortion of the octahedral coordination geometry of Cu^2+^ [68]. Prasad et al. prepared novel CPs {[Zn_2_(bdcppi)(dmf)_3_]·6DMF·4H_2_O}_n_(SNU-51) using Zn^2+^ and N,N’-bis(3,5-dicarboxyphenyl)pyromellitic diimide (H_4_BDCPPI) [69]. The Zn^2+^ in SNU-51 was ion-exchanged with exogenous Cu^2+^ and retained the PtS-type mesh structure. A microporous Mn-based MOF termed MnMnBTT (BTT = 1,3,5-benzenetetrazolium) was ion-exchanged by three ions i.e., Fe^2+^, Cu^2+^, and Zn^2+^, and the relative cation occupancy of different metal ions in the MOFs was successfully determined with multi-wavelength anomalous X-ray dispersion [70]. The results suggested that Cu^2+^ and Zn^2+^ exhibited excellent ion exchange performance at the C_4v_ site after ion exchange with an occupancy rate of 88.8% and 74.8%, respectively, much higher than Fe^2+^ with an occupancy rate of 19.5%.

Moreover, the Chen group synthesized nickel-based CPs and further introduced Co^2+^ through a cation exchange process to construct NiCo bimetallic CPs (2,5-dihydroxyterephthalic acid as the ligand) [71]. The experimental results showed that Co^2+^ doping can activate the electrochemical activity of Ni^2+^ in CPs, promote ion/electron diffusion, and reduce the polaron migration barrier, thereby improving the reversible capacity of the cell and structural stability during repeated cycling. Kim and co-workers prepared the first Ti (IV) analog of the robust UiO-66(Zr) framework using an ion exchange process [72]. The experimental results indicated that the amount of exchanged Ti^4+^ depended on the type of metal salt, with TiCl_4_ (THF)_2_ (THF = tetrahydrofuran) having the highest level of substitution while TiBr_4_ has the lowest. Even highly robust MOFs such as UiO-66(Zr), MIL, and ZIF can be fabricated by means of ion-exchange to prepare new functional materials that are currently unavailable through other synthetic methods.

#### 2.2.2. Seed Methods

The principle of the seed method is to utilize two CPs with similar lattice parameters to assemble core-shell bimetallic CPs by epitaxial growth. The matching of the crystal lattice ensures the continuous connection of pores at the crystal interface and the altered crystal structure affects the mobility as well as diffusion of the adsorbent. Furukawa et al. synthesized core-shell bimetallic CPs single crystals by seed epitaxial growth for the first time and determined the structural relationship between the shell and the core utilizing surface XRD analysis [73]. The analytical results revealed that the bimetallic MOFs with the core-shell structure consist of [{Zn_2_(ndc)_2_-(dabco)}_n_] as the core crystals and [{Cu_2_(ndc)_2_(dabco)}_n_] as the shell crystals (ndc = 1,4-naphthalenedicarboxylate, dabco = diazabicyclo[2.2.2]octane). It is essentially impossible to synthesize these two components with opposite core-shell compositions because the core crystals can be grown into single crystals with a cubic morphology, while the shell crystals can only be obtained as microcrystalline powders. In addition, bimetallic ZnZr-MOF with core-shell structure was also fabricated by seeding method and employed as an aptamer sensor platform for the detection of cancer marker protein tyrosine kinase-7 (PTK7) [74]. The results demonstrated that the crystal structure and surface functional groups of the bimetallic MOFs can be modulated by changing the order of addition between metal precursors and organic ligands. The ZnMOF-on-ZrMOF hybridized material presented a hierarchical leaf-like structure, while ZrMOF-on-ZnMOF showed a multilayer nanosheet structure.

## 3. Biosensing Applications of Bimetallic CPs

Due to the synergistic effect of different metals, bimetallic CPs have the advantages of tunable porosity, flexible luminescence, and high electrocatalytic activity, which make them versatile materials with wide applicable potentials and promising prospects in biosensing. Meanwhile, the doping of the second metal ions in unstable SBUs can greatly improve the stability of CPs, which greatly solves the disadvantage of the instability of CP materials in storage and use [75,76]. In addition, some studies have pointed out that when two or more metal ions are embedded in the structure of CPs, the bio affinity of CPs also increases, which is more favorable for application in living systems [77]. We review the work on the applications of bimetallic CPs in biosensing and biomedicine over the last five years including the detection of metal ions, small biomolecules, nucleic acids, and detection based on immune response.

### 3.1. Detection of Metal Ions

Metal ions exist widely in living organisms and are involved in many key physiological processes such as material transport, energy conversion, information transmission, and metabolic regulation. When metal ions are deficient or in excess, many health problems ensue [78]. Fe^3+^ and Cu^2+^ are involved in regulating body homeostasis and determining cellular functions, disturbances in their concentration levels in the body can lead to organ loss and serious diseases. Moreover, heavy metal ions of Hg^2+^, Pb^2+^, Cd^2+^, etc. accumulated in the human body through the food chain would lead to the occurrence of several serious diseases. Therefore, the exploration of reliable means of detecting metal ions with high sensitivity and selectivity is of great importance.

#### 3.1.1. Detection of Fe^3+^

**Figure 3:**
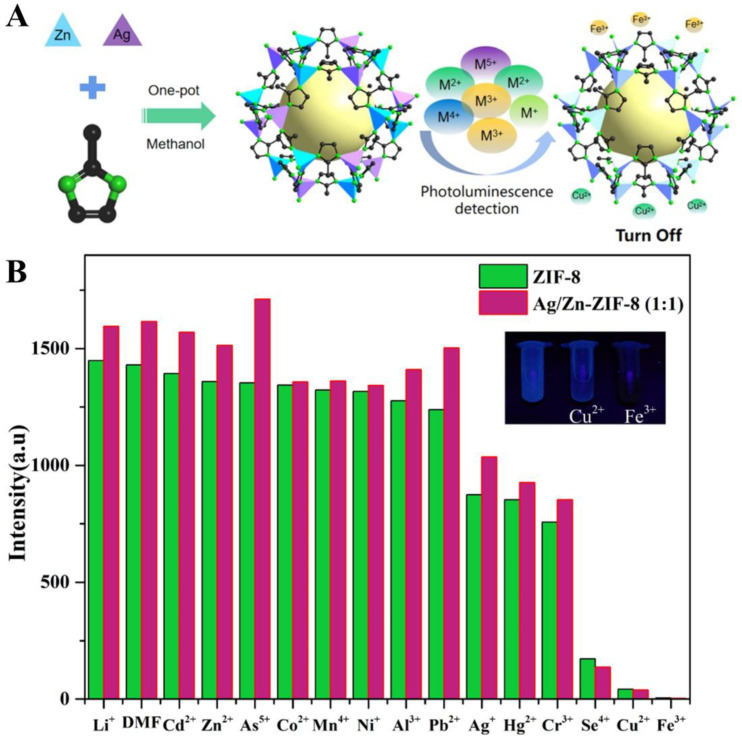
(**A**) Schematic illustration of fabrication of Ag/Zn-ZIF-8 and its direct utilization for the detection of Fe^3+^ and Cu^2+^. (**B**) Fluorescence properties of Ag/Zn-ZIF-8 (1:1) toward various cations in DMF solution (λ_ex_ = 280 nm). Reproduced with permission from Ref. [84]. Copyright 2022, Elsevier.

Iron(III) (Fe^3+^), a metal ubiquitous in the environment and in biological systems, plays an integral role in a variety of important cellular functions such as oxygen delivery in hemoglobin, oxygen metabolism, and electron transport processes in the synthesis of DNA and RNA [79]. Inadequate or excessive intake of Fe^3+^ can lead to serious chronic diseases. Therefore, it is of great significance to monitor the concentration of Fe^3+^ in the environment as well as in the human body for early diagnosis of many diseases [80,81]. Song and co-workers reported a bimetallic Eu_0.6_Tb_0.4_ MOF which possessed the maximum KSV (KSV = kinetic quenching constant) quenching constant for Fe^3+^, approximately 36.6% higher than that of monometallic Eu MOF. Furthermore, a metal-centered induced structural transition from EuMOF-1 to TbMOF-2 was achieved by changing the ratio of Eu to Tb [82]. Geng et al. successfully synthesized a series of bimetallic Cd/Zr-UiO-66 materials for fluorescence quenching probes of trace Fe^3+^ and fluorescence turn-on probes of trace As^5+^ by one-pot methods [83]. Experimental analysis showed that the fluorescence quenching of Fe^3+^ was mainly due to the competitive absorption of the excitation source and resonance energy transfer (RET), while the fluorescence enhancement of As^5+^ was mainly due to the absorbance-caused enhancement (ACE) mechanism. The fluorescent probe has good selectivity and high sensitivity, and it can maintain high luminescence stability under a variety of extreme environments. Furthermore, the same research group also developed the bimetallic Ag/Zn-ZIF-8 with strong photoluminescence properties using the one-pot method for sensing traces of Fe^3+^ and Cu^2+^ (Figure 3A) [84]. The obtained bimetallic Ag/Zn-ZIF-8 showed excellent fluorescence burst response to Fe^3+^ and Cu^2+^ with high selectivity and sensitivity, as well as good immunity to interferences with the detection limits of 3.9 μM and 6.7 μM, respectively (Figure 3B). The mechanism of the luminescence burst was investigated in detail. The resonance energy transfer (RET) and competing absorption of the excitation source from the interaction of 2-methylimidazole with Fe^3+^ and Cu^2+^ in the ZIF-8 framework may have led to efficient fluorescence bursting.

#### 3.1.2. Detection of Cu^2+^

Copper(II) (Cu^2+^) is an essential ion in biometabolic systems such as various redox processes and enzyme catalysis in living organisms. However, disturbances in the concentration of Cu^2+^ in the body can often lead to serious illnesses, such as blood and neurological disorders due to Cu^2+^ deficiency, gastrointestinal disorders, liver and neurological damage, and other illnesses due to Cu^2+^ excess [85]. Therefore, the concentration of Cu^2+^ in the environment and in humans should be strictly controlled and measured. Wu et al. synthesized the green light-emitting bimetallic 3D framework material [MgZn(1,4-NDC)_2_(DMF)_2_](1,4-NDC = 1,4-naphthalene dicarboxylic acid) [86]. Interestingly, the material showed blue fluorescence after milling, which was the first reported magnesium-based bimetallic MOF with mechanoresponsive fluorescence. The milled blue-emitting CP material maintained the same framework structure as before milling. As a result, this material can not only produce a fluorescence-quenching effect on Fe^3+^ but also act as an effective luminescent detector of CS_2_ and some nitro-explosive compounds. Peng and co-workers reported that Ti^3+^ functionalized Tb^3+^@UiO-66-(COOH)_2_ was developed as an excellent luminescent probe [87]. This probe can visually monitor Cu^2+^ in aqueous media through fluorescence quenching effect and has the advantages of high selectivity and sensitivity, wide linear concentration range (0–200 μM), low detection limit (0.23 μM) and fast response time (within 1 min). The mechanism of quenching luminescence is the coordination of Cu^2+^ with the free carboxylic acid group of Tb^3+^@UiO-66-(COOH)_2_, which leads to a decrease in the energy transfer efficiency from the ligand to the Tb^3+^ ions and thus quenches the fluorescence.

#### 3.1.3. Detection of Other Metal Ions

Along with the rapid industrial development in modern society, heavy metal pollution in the environment is growing at an alarming rate [88]. Excess heavy metals will gradually accumulate in the food chain and eventually pose a serious threat to human health. Heavy metal-induced toxicity and carcinogenicity involve many mechanistic aspects. For instance, lead (Pb) poisoning can cause serious damage to the human nervous, skeletal, and immune system [89]. Chromium (Cr) is considered one of the most carcinogenic elements and can cause lung cancer, prostate enlargement, and other diseases [90]. Mercury (Hg), the heavy metal that readily accumulates in the food chain, would damage the respiratory system as well as the central and peripheral nervous systems, exceeding the safe levels in the human body [91]. Even excessive accumulation of zinc (Zn), one of the essential trace elements, can lead to zinc poisoning and acute renal failure [92]. Therefore, the development of rapid detection methods for heavy metal ions in the environment and the human body is an urgent need. Chen and co-workers used a modified sodium hydroxide mediated method to synthesize ZIF-67 doped with Mn^2+^, Fe^3+^, and Ni^2+^, the resulting product was used to construct an electrochemical sensing interface for detection of Hg^2+^ through square wave anodic stripping voltammetry [93]. The results showed that the electrochemical signals for Hg^2+^ detection were enhanced when ZIF-67 was doped with metal ions compared to pure ZIF-67. Among them, the Fe^3+^-modified ZIF-67 showed excellent performance in the trace detection of Hg^2+^. Its sensitivity (41.5 μA/μM) and LOD (7.82 nM) (LOD = limit of detection) exceeded the requirements of the World Health Organization’s analytical method for the detection of Hg^2+^ in drinking water. The Sun group reported a novel BiCu MOF-derived carbon film encapsulating BiCu alloy nanoparticles (BiCu-ANPs) integrated into an electrochemical sensing system for real-time on-site ultrasensitive detection of Pb^2+^, Cd^2+^, and Zn^2+^ in different human biofluids and environmental water [94]. The results revealed that the novel bimetallic material significantly improved the electrocatalytic activity and stability, which was attributed to the strain and electronic effects induced by the encapsulated structure of the hybrid metal and carbon framework. As a result, the constructed electrochemical sensing system had multiple active sites, good electrical conductivity, fast enrichment time and high stability, which consequently improved the overall performance and reliability of the sensing system.

### 3.2. Detection of Small Molecules

Precise monitoring and rapid screening of disease-marking small molecules in blood, saliva, sweat, urine, and tears are critical for early prevention of many diseases [95,96,97]. Compared to monometallic CPs, bimetallic CPs with uniform distribution of two metal elements and well-controlled morphology exhibit superior luminescence, and catalytic, conductive, and energy-converting properties through the synergistic effect of multiple components. In practical applications, bimetallic CPs are often combined with other nanomaterials (for example, nanoparticles, field-effect transistors, and natural enzymes) to form composite biosensors for rapid and sensitive detection of small molecules as disease markers in complex and variable sensing environments [98,99,100].

#### 3.2.1. Detection of Glucose

Diabetes mellitus is intricately associated with blood glucose levels, sensitive and rapid detection of blood glucose is important for early monitoring and timely treatment of diabetes mellitus [101]. Assays using bimetallic CPs for glucose detection are mainly divided into non-enzymatic electrochemical assays and peroxidase-mimicking assays.

**Figure 4:**
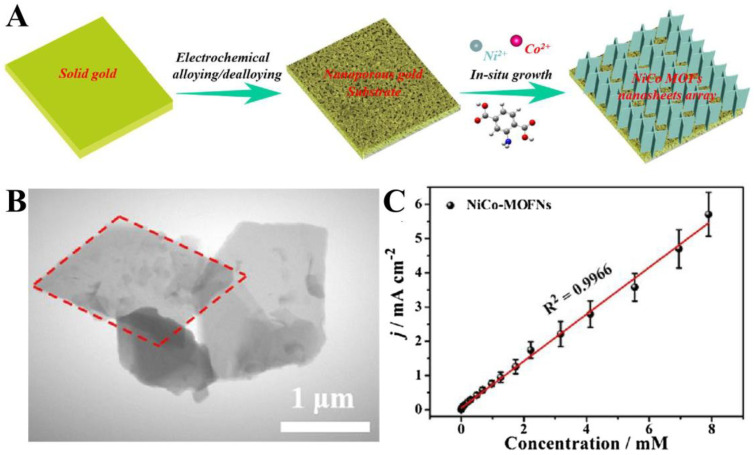
(**A**) Schematic illustration for the preparation of vertical NiCo-MOF nanosheets array. (**B**) TEM image of vertical NiCo-MOF nanosheets. (**C**) Amperometric response calibration curve for glucose detection by the constructed electrode. Reproduced with permission from Ref. [102]. Copyright 2019, Elsevier.

Due to the synergistic effect of the two metals, bimetallic CPs possess superior electrocatalytic properties and are widely used as efficient electrocatalysts. The sensing mechanism is that strong electronic interactions between the two ions and the ligand greatly enhance the electrochemical catalytic oxidation of glucose to achieve signal amplification. Li et al. successfully prepared novel vertical 2D NiCo bimetallic organic framework (NiCo-MOF) parallelogram nanosheet arrays on the nanoporous gold surface using a bottom-up approach (Figure 4A) [102]. The vertical alignment feature of the MOF nanosheets exposed more electrocatalytically active sites and facilitated the charge transfer for electrochemical reactions (Figure 4B). The 2D NiCo-MOF nanosheet array electrode has excellent glucose detection performance with a linear range of 1 μM~8 mM, fast response time of less than 1 s, sensitivity of 0.6844 mA·mM^−1^cm^−2^ and LOD as low as 0.29 μM (Figure 4C). Zha and co-workers used a simple solvothermal method to synthesize 3D nanoflower-like bimetallic NiCo-MOF consisting of 2D nanosheets as electrode materials for micro-supercapacitors and sensing materials for glucose sensors [103]. The designed enzyme-free glucose electrochemical sensor has a high sensitivity of 0.31 μA·μM^−1^ and a low LOD of 10 μM for sensitive and rapid detection of glucose in blood. In addition, a wearable non-invasive sensor system was developed by integrating the glucose sensor with a micro-supercapacitor (MCS) on a flexible polyethylene terephthalate (PET) substrate, which has significant potential in the field of non-invasive sweat glucose detection.

CPs, as a new type of mimetic enzyme, are widely used in the field of biosensing due to their low cost, simple preparation, high stability, and good recyclability compared with natural enzymes [104]. Bimetallic CPs contain multiple active sites, which drastically improve the catalytic activity more than monometallic CPs with a single site. In addition, porous MOF materials are likewise immobilized loading platforms for many natural enzymes to improve the stability of natural enzymes in use [105]. Bimetallic CPs are often used together with natural enzymes to form composite biosensors for sensitive and rapid detection of glucose. The Duan group prepared a novel glucose biosensor based on bimetallic Ni/Cu-MOFs (GOD-GA-Ni/Cu-MOFs-FET, GOD = glucose oxidase, GA = glutaraldehyde, FET = field-effect transistor) by a simple one-step hydrothermal method [106]. Due to the dual role of Ni/Cu-MOF as a peroxidase mimic and a protective coating, the obtained multi-enzyme system possesses both peroxidase-like activity and the biological activity of natural enzymes. Accompanied by the properties of segmental linearity over a wide range of 1 μM–20 mM, high sensitivity (26.05 μA·cm^−2^mM^−1^), and low LOD (0.51 μM) in the low concentration range of 1~100 μM, the glucose sensor has the advantages of high specificity, good reproducibility, and good short-term stability. In addition, the Fe_3_Ni-MOF nano-enzymes synthesized by Mu and co-workers showed excellent peroxidase-like catalytic activity and the mechanism was investigated by cyclic voltammetry and electron spin resonance (ESR) [107]. The results suggested that the electron transfer from TMB (TMB = 3,3′,5,5′-tetramethylbenzidine) to H_2_O_2_ was enhanced by the incorporation of Ni into Fe-MOF. At the same time, the redox capacity of Fe_3_Ni-MOF was improved due to the enhanced electron transfer between Fe^2+^ and Fe^3+^, which subsequently promoted the generation of hydroxyl radicals (·OH), and thus the peroxidase-like activity. Together, these two mechanisms significantly enhanced the peroxidase-like activity of Fe_3_Ni-MOF. Taking advantage of the excellent peroxidase-like activity of Fe_3_Ni-MOF, a biosensor (Fe_3_Ni-MOF/GOx) (GOx = glucose oxidase) for the detection of glucose was prepared by adsorbing GOx onto Fe_3_Ni-MOF. A one-step colorimetric method was established and successfully applied to the detection of glucose in human serum samples.

#### 3.2.2. Detection of Dopamine (DA)

Dopamine (DA) is an important catecholamine neurotransmitter widely distributed in the central nervous system, cardiovascular system, hormones, and kidneys [108]. Abnormal DA concentrations often lead to neurological disorders such as schizophrenia and Parkinson’s disease. Therefore, the establishment of an analytical method for the sensitive and rapid detection of DA is essential for disease assessment. In the physiological environment, DA usually coexists with ascorbic acid (AA) and uric acid (UA), whose oxidation potentials are very close to each other, resulting in poor selectivity, high LOD, and difficult detection. The diversity of types and arrangements of active metal sites in bimetallic CPs has been proved to be more beneficial for the transfer and exchange of electrons in the system, thereby achieving the purpose of regulating the type and number of active sites in CP materials. Moreover, multiple ions tend to have a synergistic promotion effect, which can remarkably enhance the catalytic activity of CPs, resulting in better electrocatalytic performance than that of the monometallic system. Duan et al. fabricated bimetallic Fe_2_Ni-MIL-88B material as an enzyme-free DA sensor employing hydrothermal methods [109]. The Fe_2_Ni MIL-88B/GCE constructed by modifying Fe_2_Ni MIL-88B onto a glassy carbon electrode (GCE) exhibited satisfactory electrochemical catalytic performance for DA, with a linear range of 1.2 μM~1.8 mM, LOD of 0.40 μM and sensitivity of 124.7 μA·mM^−1^ cm^−1^. The electrochemical sensor demonstrates acceptable specificity, stability, and reproducibility in the analysis of real samples. Ma and co-workers adopted a simple surfactant-assisted method to synthesize 2D Co/Zn porphyrin (Co/Zn-TCPP) [TCPP = tetrakis(4-carboxyphenyl) porphyrin] MOF nanomaterial known as Co_25_Zn_75_-TCPP and constructed a new DA sensing method based on this material [110]. The doping of metal ions not only improved the chemical environment of the original pores but also multiplied the type and spatial arrangement of the MOF active sites, which was conducive to electron transfer and exchange with DA. The active centers of the two metal ions were synergistically promoted, resulting in a significant enhancement of the electrocatalytic activity of the MOF. The constructed sensor had a linear range of 5 nM–177.8 μM with a detection limit of 1.67 nM (S/N = 3) at a potential of 0.1 V and exhibited promising selectivity for DA. The Shuang group has fabricated CoNi-MOF@ERGO (ERGO = electrochemically reduced graphene oxide) composite combining CoNi-MOF with ERGO on a glassy carbon electrode by electrochemical reduction method [111]. Co^2+^ and Ni^2+^ in the composites served as active sites to accelerate electron transfer and 2-methylimidazole as adsorption sites to enhance the enrichment of DA. The results indicated that the synergistic effect of CoNi-MOF and ERGO enhanced the catalytic performance with good selectivity for DA detection. In addition, the sensor was able to detect DA in a real environment of human serum samples with a satisfactory recovery range.

#### 3.2.3. Detection of Hydrogen Sulfide (H_2_S)

Hydrogen sulfide (H_2_S) is a signal-regulating molecule in the central nervous system and has been classified as the “third gas transmitter” after carbon monoxide (CO) and nitric oxide (NO). H_2_S is closely related to physiological activities such as cell growth, vasodilatation, diagnosis, inflammation, and renal function, its abnormal concentration levels are highly correlated with persistent diseases [112]. Therefore, it is crucial to construct reliable biosensors for rapid and sensitive detection of H_2_S. Luminescent bimetallic CPs have received considerable attention due to their versatile and tunable optical properties [113]. The fluorescence properties of bimetallic CPs are determined by the energy transfer between the metal ions and the ligands. The secondary doping of the metal ions provides more versatility in the sensing process, making bimetallic CPs a superior sensing and detection platform. Zhu and co-workers reported a new bimetallic MOF, Fe_x_Al_1-x_-MIL, for the detection of H_2_S in aqueous systems [114]. In this bimetallic MOF, two transition metals with low cost and abundant reserves (Fe^3+^ and Al^3+^) were used as metal nodes, while 2-aminobenzene-1,4-dicarboxylic acid (BDC-NH_2_) was used as the bridging ligand. The presence of the -NH_2_ group endowed the Al-based MOF to exhibit strong blue fluorescence. Owing to the transposition of partial Fe^3+^ with Al^3+^ in the Fe^3+^-doped Al-MIL-NH_2_, a strong ligand-to-metal charge transfer (LMCT) between Fe^3+^ ions and BDC-NH_2_ ligands was generated, making the bursting effect within Fe_x_Al_1-x_-MIL much effective. When Fe_0.05_Al_0.95_-MIL was exposed to H_2_S (0–38.46 μM), the fluorescence intensity showed a good linear correlation with H_2_S concentration, indicating that the bimetallic MOF could be used for selective and sensitive detection of H_2_S. The mechanism of fluorescence enhancement in this system was unveiled. In the H_2_S sensing process, Fe^3+^ in Fe_0.05_Al_0.95_-MIL after H_2_S treatment was “pulled out” and captured by S^2−^, then the quenching effect was lifted and the released BDC-NH_2_ ligand acted as a true fluorophore, which contributed to the fluorescence enhancement. Huang group reported a simple and robust strategy based on bimetallic Ni-Co-MOF with poly(3,4-ethylenedioxythiophene) (PEDOTs) and poly(ophenylenediamine) (PoPDA) to fabricate a real-time H_2_S sensor [115]. The PEDOTs@Ni-CoMOF/GCE H_2_S sensor showed an enhanced catalytic performance with a concentration range of 1 nM to 250 µM, low LOD (0.186 nM), and high sensitivity (7.29 μA μM^−1^ cm^−2^). The sensing system utilized an endogenous sensor to continuously measure the H_2_S levels in organic donors and live cells, which provided a new research idea for the electrochemical detection of H_2_S in physiological and pathological processes.

#### 3.2.4. Detection of Uric Acid (UA)

Uric acid (UA) is an important product of purine metabolism, deviations in UA levels are suggestive of a variety of diseases, such as hyperuricemia, gout, Lesch–Nyan disease, etc. [116]. Therefore, the detection of UA is vital for health monitoring and disease diagnosis. Han and co-workers reported the first MOF nano-enzymatic source proportional fluorescent UA sensor based on the Fe_3_Ni-MOF-NH_2_-propelled UA/uricase/phthalimide tandem catalytic reaction [117]. Unlike previous reports, this work exploited both the peroxidase-like and fluorescence properties of Fe_3_Ni-MOF-NH_2_. In the absence of UA, only blue fluorescence of MOF at 430 nm was observed, whereas the addition of UA would trigger the catalytic reaction of UA/uricase catalyzed by the MOF mimetic enzyme, and the generated H_2_O_2_ would oxidize o-phenylenediamine to the highly luminescent 2,3-diaminophenazine (DAP) (emission wavelength = 565 nm). Coincidentally, the fluorescence of MOF can be quenched by DAP through an internal filtering effect, resulting in a lower I_430nm_ value and a higher I_565nm_ value. Therefore, a ratiometric fluorescence sensor for detecting UA was constructed by monitoring the opposite fluorescence changes described above. The LOD of this sensor was 24 nM, much lower than most previous reports. In addition, intelligent portable sensing of UA was conveniently achieved using the same sensing system and smartphone-based RGB (RGB = red, green, and blue) analysis. Furthermore, molecular antilogical calculations initiated by nanoenzyme catalysis were performed. This work not only provides a cost-effective, portable, and solid prototype for a highly sensitive and reliable MOF mimetic enzyme-based multifunctional ratiometric fluorescent biosensor to detect UA but also opens up novel frontiers for future logic-engineered POCT (POCT = point of care testing) biomarker analysis and disease diagnosis.

#### 3.2.5. Detection of Methylglyoxal (MGO)

Methylglyoxal (MGO) contains a reactive dicarbonyl group and is produced mainly by glycolysis in living cells. Studies have shown that MGO causes protein misfolding and unfolding in the lumen of the endoplasmic reticulum (ER) as well as abnormalities in calcium metabolism [118]. Also, the disturbances in its concentration are strongly associated with obesity, diabetes, and Alzheimer’s disease (AD) [119]. Detection of MGO in vivo is important for diagnosis and even study of the pathological process of its corresponding diseases. Zheng et al. have designed and developed two novel composites based on Tb(III) and Yb(III) functionalized Cu(II) CPs with enhanced thermal and water stability as well as fascinating fluorescence properties [120]. Among them, Tb@Cu-Hcbpp (Hcbpp = 1-(4-carboxylbenzyl)-3-(pyrzin-2-yl) pyrazole) exhibits broad ligand-centered emission and weak typical Tb^3+^ ion emission, which can be used as an excellent ratiometric fluorescent sensor for the human N,N-dimethylformamide (DMF) metabolite N-methylformamide (NMF) (LOD = 0.02 μM). In addition, Yb^3+^ ions can be doped into Tb@Cu-Hcbpp to obtain multi-doped luminescent CP materials with enhanced luminescence properties. In particular, the fluorescence enhancement intensity of Tb_0.85_Yb_0.15_@Cu-Hcbpp is almost 9.6 times higher than that of the pure Tb^3+^ system, and it also has a high fluorescence burst efficiency for methylglyoxal (MGO), which can be used for the sensitive detection of MGO (LOD = 0.25 μM). Based on these results, the developed biosensor has been successfully applied to detect NMF and MGO in urine and serum samples with satisfactory results.

### 3.3. Detection of Biomacromolecules

Detection of biomacromolecules is important for health monitoring and early diagnosis of diseases. A large number of monometallic CPs have been applied to detect biomolecules (for example, nucleic acids, alkaline phosphatase, extracellular vesicles, carcinoembryonic antigen, immunoglobulins G, etc.), but the performance of the assays was unsatisfactory [95]. Bimetallic CPs comprised of two kinds of metal ions, such as Cu^2+^ and Zn^2+^, have shown superior performance in the detection of biomolecules, such as nucleic acids and peptides, due to the synergistic effect of the bimetals, which are gradually applied for sensitive, rapid and selective detection [121].

#### 3.3.1. Detection of Nucleic Acid

**Figure 5:**
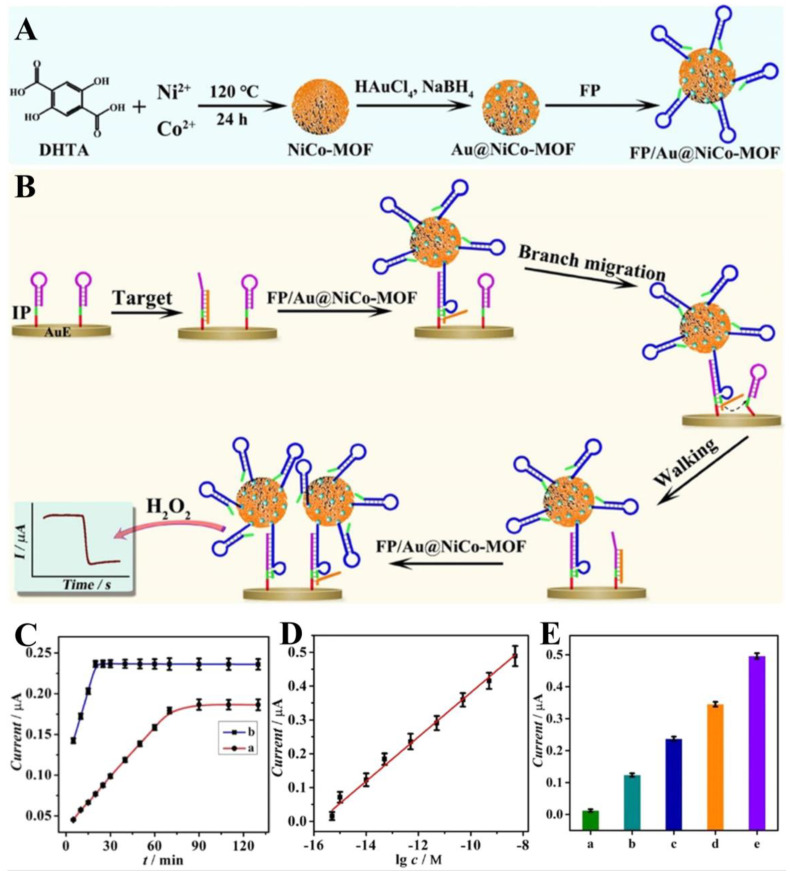
(**A**) Schematic illustration of the fabrication process for FP/Au@NiCo-MOF. (**B**) Mechanism for constructing a wedge-shaped DNA Walker for rapid and sensitive detection of methylated target DNA. (**C**) Effect of incubation time on the responses of the traditional DNA walker (red line) and this wedged DNA walker (blue line). (**D**) Linear relationship between the current values and the logarithmic concentration of methylated DNA (from 0.5 fM to 5 nM). (**E**) Current responses of human serum samples containing (a) 0 fM, (b) 10 fM, (c) 500 fM, (d) 50 pM, and (e) 5 nM. Reproduced with permission from Ref. [125]. Copyright 2023, American Chemical Society.

Different kinds of miRNAs, such as miRNA-126, miRNA-224, and miRNA-30d-5p, are cancer promoters overexpressed in lung cancer tumor cells [122]. The selective and sensitive detection of different kinds of miRNAs is of great significance. Bimetallic MOFs have emerged as promising materials for the construction of electrochemical biosensors, owing to their enhanced electrochemical activity compared to conventional monometallic MOFs [123]. The porous structure with adjustable voids enriches free miRNAs and ensures rapid and sensitive detection. The Du group formulated a bimetallic CoNi-MOF and employed it to construct biosensors for sensitive and selective detection of miRNA-126 [124]. The mixed coordination of the metal centers of Co and Ni with carboxyl and pyridyl groups greatly enhanced the electron transfer and amplified the electrochemical signals, thus eliminating the need for electrochemical indicators and blockers to eliminate the non-specific adsorption between miRNA-126 and CoNi-MOF. The assay results demonstrated that the biosensor had an ultra-low LOD of 0.14 fM in the range of 1 fM~10 nM, which was suitable for the selective, sensitive and reproducible detection of miRNA-126. Dou et al. built an efficient DNA walker attached to a stable AuNP-coated bimetallic MOF electrocatalyst for H_2_O_2_ reduction to detect DNA methylation (Figure 5A) [125]. The wedge-shaped segments on the tracks were designed to be continuously complementary to the target methylated DNA, thus inhibiting its separation from the tracks. The bimetallic MOF carrying the fuel strand not only promoted the gradual movement of the target strand but also acted as an efficient catalyst for H_2_O_2_ reduction on the sensing platform (Figure 5B). After implementing the aforementioned innovative design, the sensor achieved sensitive monitoring of methylated DNA within 20 min (Figure 5C) with a detection limit as low as 200 aM (Figure 5D). Moreover, the serum detection exhibited acceptable recoveries ranging from 97.9 to 106.5%, displaying the potential application of this biosensor for real sample detection (Figure 5E).

#### 3.3.2. Detection of Alkaline Phosphatase (ALP)

Alkaline phosphatase (ALP) is a membrane-bound enzyme with the ability to catalyze dephosphorylation. This hydrolase is also involved in the transduction of in vivo signals and the regulation of intracellular growth and apoptotic processes [126]. Overexpression of ALP is usually associated with liver and bone disease (osteoblastic bone cancer, Paget’s disease, and osteochondrosis), whereas under-expression of ALP leads to hypophosphatasia [127]. Therefore, it is of utmost importance to develop a simple and accurate method for the quantitative determination of ALP expression. Wang et al. have prepared red-illuminated Tb-GMP-Eu CPs (GMP = Guanine monophosphate) for the detection of ALP activity via a one-pot method [128]. The red luminescence of Tb-GMP-Eu CPs arises from the synergistic interaction of the phosphate groups in the GMP ligand with Tb^3+^ and Eu^3+^. In the presence of ALP, the phosphoryl group in the GMP ligand is catalytically broken down, leading to an interruption in the energy transfer from Tb^3+^ to Eu^3+^ thereby causing a fluorescence burst in the Tb-GMP-Eu CPs. These CPs have the advantages of simple synthesis, good biocompatibility, and photostability, as well as high sensitivity and selectivity response to ALP in the concentration range of 0.05~20 U·L^−1^, with an LOD of 0.004 U·L^−1^. Furthermore, real-world sample testing presented that the biosensors could be successfully employed in the evaluation of ALP inhibitors and the determination of ALP in serum.

#### 3.3.3. Detection of Carcinoembryonic Antigen (CEA)

Carcinoembryonic antigen (CEA) is an important cancer biomarker for the monitoring and diagnosis of colon, breast, ovarian, colorectal, and cystic adenocarcinomas [129]. CEA levels in healthy individuals are usually at the ng·mL^−1^ level, and consequently, the construction of an ultrasensitive and accurate CEA assay is of great significance for early screening of cancer. According to past reports, current detection techniques (fluoroimmunoassay, electrochemiluminescence immunoassay, radioimmunoassay, plasmonic nanoimmunosensor assay, and enzyme-linked immunoassay) usually made use of antibodies as recognition elements [130,131,132,133,134]. Antibodies can specifically recognize the target analyte, but many of them have the disadvantages of immunogenicity, toxicity, and high cost, which limit their wide clinical application. The Du group synthesized a Zr-MOF containing an n-carboxylic acid-based ligand (2,2′-bipyridine 5,5′-dicarboxylic acid, bpydc), in which Co ions were introduced to form a bimetallic ZrCo-MOF [135]. Aptamer chains targeting CEA were anchored to ZrCo-MOF to prepare biosensors capable of sensitive and selective detection of CEA. ZrCo-MOF has a large specific surface area, tunable porous structure, and good biocompatibility, offering more active sites for the immobilization of aptamers. The aptamer chains can be anchored to the surface of the ZrCo-MOF-modified electrodes through various effects such as Zr-O-P bonding, Co-N coordination, π-π* superposition, and van der Waals forces, which are more tightly bonded than single metal Zr-MOFs. The doping of Co^2+^ in the MOF skeleton enhanced the electrochemical activity of the whole sensor, which could effectively improve the sensitivity of detecting CEA. The ZrCo-MOF-based aptasensors exhibited excellent sensitivity, selectivity, stability, reproducibility, and utility for real human serum samples, demonstrating the potential to be applied to biosensing and clinical diagnosis of cancer.

#### 3.3.4. Detection of Extracellular Vesicles (EVs)

Extracellular vesicles (EVs), which are widely present in biological fluids, are closely associated with immune responses, cancer metastasis, and cardiovascular or central nervous system-related diseases [136]. However, the extremely low concentration of EVs in biological samples makes the detection of EVs by conventional methods challenging and limiting [137]. Therefore, simple and sensitive rapid EV detection techniques need to be developed for early diagnosis of diseases and health monitoring. Jiang and co-workers reported a bimetallic Fe/Co-MIL88(NH_2_) with excellent peroxidase catalytic activity and superior stability due to the abundant active site and synergistic effect between Fe^3+^ and Co^2+^ [138]. Subsequently, Fe/Co-MIL88(NH_2_) was modified by GOx, triggering a cascade enzymatic reaction for the highly sensitive detection of EVs. The cascade enzymatic reaction of Fe/Co-MIL88(NH_2_) with GOx can achieve the detection of EVs as low as 7.8 × 10^−4^ particles/mL, and this detection limit showed two orders of magnitude lower than that of horseradish peroxidase (HRP). The accuracy and high recoverability of the biosensor as evidenced by actual sample testing results illustrated its potential for clinical analysis and early disease diagnosis.

#### 3.3.5. Detection of Immunoglobulin G (IgG)

Human immunoglobulin G (IgG) is the primary antibody present in human blood and plays a crucial role in the immune system by recognizing and defending against foreign antigens [139]. The potential for developing sensitive, rapid, and selective IgG sensing assays is crucial for vaccine development, early diagnosis of immune disorders, and the advancement of therapeutic approaches. Conventional methods such as enzyme-linked immunosorbent assay (ELISA) and western blotting require sophisticated specialized equipment as well as high costs, thereby leading to an urgent need to develop simple, rapid, and low-cost assays [140]. Bimetallic CPs have distinct advantages of flexible tunability, large specific surface area as well as high sensitivity, their conductivity is greatly improved compared to monometallic CPs due to the synergistic effect of bimetallic ions. The ultra-sensitive electrochemical detection method developed based on bimetallic CPs has attracted much attention in the field of IgG detection. Ravipati et al. reported bimetallic Ni/Co-MOF modified nickel foam electrodes for IgG detection utilizing solvothermal methods [141]. When IgG molecules were introduced to the electrode surface, they interacted with the redox active sites on the Ni/Co-MOF, altering the electron transfer kinetics and charge distribution resulting in measurable changes in current or potential. The prepared Ni/Co-MOF/NF sensor exhibited high sensitivity (28 µA cm^−2^ mol^−1^) and high selectivity at trace levels (30 fM to 10 nM), confirming the potential for the detection of Ig molecules in clinical diagnostics and biomedical research.

### 3.4. Detection of Drug Molecules

Drug molecules including antibiotics, anticancer and non-steroidal anti-inflammatory drugs play a pivotal role in life activities and medical therapy. Determination of the presence or concentration of specific drugs in biological fluids (for example, serum) is essential for determining the physiological and clinical manifestations of drugs [142]. Meanwhile, residues from the extensive use of antibiotics have significant impacts on agricultural products, ecosystems, and human health [143]. Moreover, drug abuse is a drug-related disorder in which drugs are taken regularly and users take them in quantities or by methods that are harmful to themselves or others [144]. Therefore, the exploitation of fast, sensitive, and specific sensors for the detection of drug molecules in the environment and in the human body is very urgent.

#### 3.4.1. Detection of Doxorubicin (DOX)

The anticancer drug doxorubicin (DOX) is a first-line treatment for breast, ovarian, thyroid, and leukemia cancers, but it is strictly considered in clinical use due to its side effects such as cardiotoxicity, myelosuppression, nausea, and alopecia [145,146]. Therefore, the development of efficient and sensitive assays to detect DOX in the human body to regulate the dose is essential in chemotherapy. Electrochemical sensors constructed on the basis of bimetallic CPs provide a selective, sensitive, and fast low-cost alternative for the detection of DOX. The Ahmadi group reported the preparation of an electrochemical sensor for the detection of DOX relying on the in situ growth of NiCo-BTC bimetallic MOFs on a glassy carbon electrode modified with conductive nitrogen-doped graphene oxide nanoribbons (NiCo-BTC MOFs/N-GONRs/GCE) [147]. The square-wave voltammetric response of NiCo-BTC MOFs/N-GONRs/GCE to DOX was significantly larger than that of NiCoBTC MOFs/GCE due to the synergistic interaction between N-GONRs and NiCo-BTC MOFs to enhance the conductivity and sensitivity. NiCo-BTC MOFs/N-GONRs/GCE achieved a sensitive and selective detection of DOX with a low LOD (6 nmol L^−1^) in the linear dynamic range of 0.01~1.0 and 1.0~80 μmol L^−1^. The high recovery rate for detecting DOX in real samples demonstrates how this novel biosensor will open new avenues for the development of bimetallic MOFs-based electrode materials with excellent conductivity.

#### 3.4.2. Detection of Enrofloxacin (ENR)

**Figure 6:**
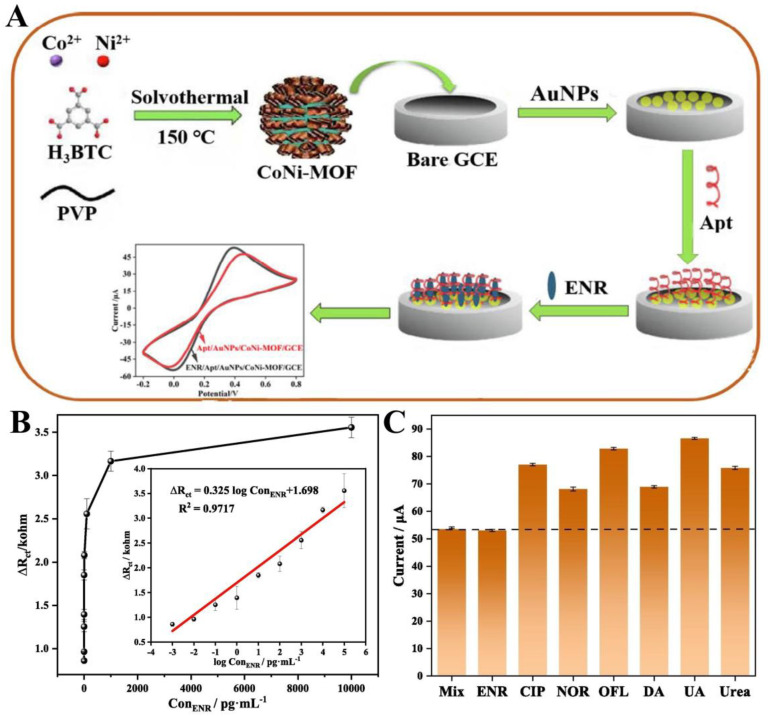
(**A**) Schematic illustration of the preparation of Apt/AuNPs/CoNi-MOF/GCE electrochemical sensor and its voltammetric response toward ENR. (**B**) Effect of different concentrations of ENR on ΔR_ct_. Inset: the calibration curves. (**C**) Peak current values of the aptasensor for interferences (10 μg·mL^−1^), ENR (100 ng·mL^−1^), and their mixture (100 ng·mL^−1^). Reproduced with permission from Ref. [150]. Copyright 2022, Elsevier.

The synthetic third-generation fluoroquinolone antibiotic enrofloxacin (ENR) is widely used for the treatment of respiratory infections in animals with Mycoplasma organisms and Gram-negative bacterial infections [148]. However, overuse of antibiotics would bring many unavoidable side effects, such as nausea, vomiting, diarrhea, headache, insomnia, and other symptoms in humans as well as animals [149]. Therefore, while antibiotics remain a key tool in the prevention and treatment of infectious diseases, levels of antibiotics in the body and the environment must be monitored and regulated. Electrochemical aptamer sensors have many advantages in the detection of antibiotics including low cost, non-toxicity, high sensitivity, and superior selectivity. Bimetallic CPs are excellent electrode materials for the construction of electrochemical aptamer sensors due to the synergistic effect of each component with better conductivity and simple modification by aptamers. Wei and co-workers fabricated a novel electrochemical aptamer sensor based on bimetallic CoNi-MOF material and gold nanoparticles (AuNPs) for the detection of ENR (Figure 6A) [150]. In this work, AuNPs were modified on CoNi-MOF/GCE by electrodeposition to improve the conductivity of the electrode material and accelerate the aptamer to be loaded onto AuNPs/CoNi-MOF/GCE by Au-S bond. The obtained AuNPs/CoNi-MOF/GCE showed a satisfactory detection ability for ENR in the linear range of 0.001–1 × 10^5^ pg·mL^−1^ with LOD as low as 0.33 fg·mL^−1^ (Figure 6B). Moreover, the electrochemical aptamer sensor showed excellent selectivity, favorable reproducibility, and high stability in the detection (Figure 6C) of ENR in real samples. It demonstrates the potential of bimetallic MOF-based electrochemical aptamer sensors to be widely applied in the field of biosensing.

#### 3.4.3. Detection of Levofloxacin (LEV)

Levofloxacin (LEV) is a fluoroquinolone antibiotic utilized for the treatment of a variety of diseases associated with infections caused by sensitive strains of bacteria [151]. Due to the poor metabolism of LEV in the human body, approximately 87% of the ingested drug can be recovered in the urine and the accumulated LEV in the environment would pose a greater risk to humans and other animals [152]. Therefore, in order to detect LEV levels in humans and the environment more rapidly, sensitively, and selectively, Deng and co-workers proposed a dual recognition and dual amplification detection strategy for levofloxacin based on a Cu/Fe-BTC MOF-modified electrode sensor [153]. After the elution of levofloxacin, a new electrochemical assay for the detection of levofloxacin was established based on the obtained recognition site with levofloxacin, which effectively excluded the interference of the enantiomer of D-ofloxacin. In addition, the synergistic effect of Cu/Fe-BTC effectively amplified the current response signal and improved the sensitivity of the sensor. The linear range of the sensor for LEV detection was 5~4000 × 10^−11^ mol L^−1^ and the LOD was as low as 2.07 × 10^−11^ mol L^−1^. The designed electrochemical sensor for the detection of levofloxacin in real samples had high recoveries (92.7~109.8%), showing great potential in antibiotic detection.

#### 3.4.4. Detection of Paracetamol

Paracetamol, also known as acetaminophen, is one of the most commonly used analgesic and antipyretic drugs. It has been widely applied as an effective treatment for relief from pain and fever [154]. In contrast to other analgesic and antipyretic agents, paracetamol does not cause direct damage to the body. However, overdosage of paracetamol would lead to the formation of a number of hepatotoxic and nephrotoxic metabolites which would in turn cause diseases such as acute hepatic necrosis [155]. Shalauddin et al. prepared FeMg MOF-BPN nanocomposites by combining FeMg MOF and black phosphorous nanosheets (BPN) for the first time, using the drop-casting method [156]. The FeMg MOF coating can effectively improve the inherent stability of BPN. The carboxylic acid groups from the MOF ligand terephthalic acid can not only effectively chelate with Fe and Mg atoms, but also bond with the BP layer through hydrogen bonding and electrostatic interactions to form a highly stable heterostructure with a higher surface area, which provides sufficient redox active sites and ensured effective binding sites for the target molecules. In the presence of paracetamol, the linear detection range of the sensor was 0.002–30 µM and 40–700 µM with sensitivity values of 23.61 µA µM^−1^ cm^−2^ and 0.94 µA µM^−1^ cm^−2^, respectively. The recoveries of paracetamol in pharmaceutical preparations and simulated blood samples were 99.56–100.60% and 99.50–101.75%, respectively, indicating the reliability of the sensor in the detection of real samples.

## 4. Biomedical Applications of Bimetallic CPs

Cancer poses a serious threat to the health of all human beings and causes millions of deaths each year [157]. For cancer treatment, traditional cancer drugs and cancer vaccines as well as emerging functional nanomaterials (light-sensitive, heat-sensitive, and microwave-sensitive materials) are effective therapies [158]. Directly administered treatments produce numerous undesirable side effects including poorer pharmacokinetics as well as biodistribution [159]. Hence, it is crucial to emphasize that drugs, vaccines, and functional nanomaterials must possess the ability to selectively target the specified cancer cells while avoiding any damage to healthy tissues [160]. An efficient, stable, and selective drug delivery system (DDS) helps in controlling drug release and correcting drug efficiency. Porous MOF materials with large drug loading capacity, good biocompatibility, and easy degradation by the human body are popular choices in DDS lately [161,162,163]. Bimetallic MOFs show more adjustable structure, better stability, superior luminescence, and even better enzyme mimicry performance than monometallic MOFs due to the synergistic effect of multiple components, which are gradually becoming more active in the front line of drug delivery and cancer therapy.

### 4.1. Bimetallic CPs Based on Anticancer Drugs

**Figure 7:**
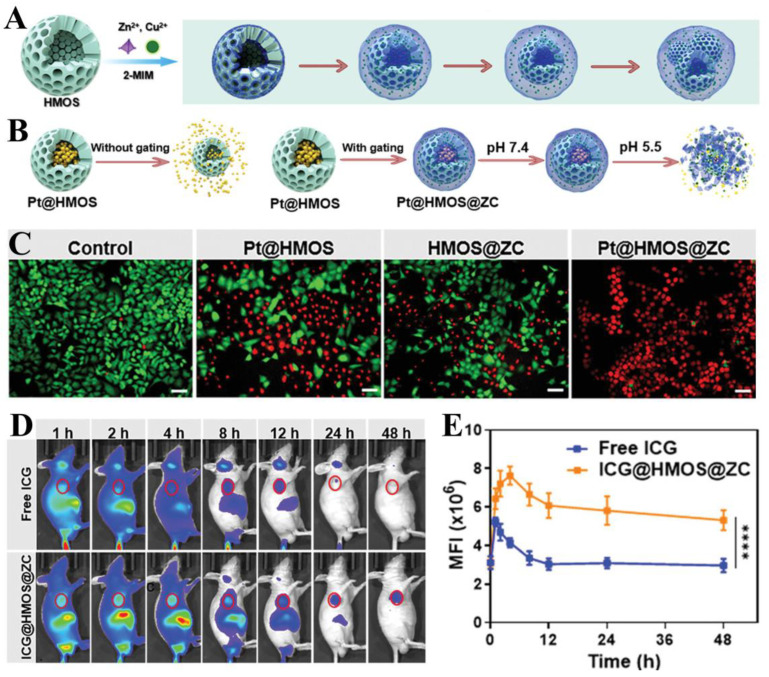
(**A**) Scheme for the synthesis of HMOS@ZC. (**B**) Schematic for Pt@HMOS without bimetallic MOF gating and Pt-based drugs release profiles of Pt@HMOS at different pH values. (**C**) Fluorescence images of Calcein AM (green, live cells) and PI (red, dead cells) co-stained A549 cells treated by different formulations for 24 h. (**D**) In vivo fluorescence images of A549-tumor-bearing mice and (**E**) quantitative mean fluorescence intensity analysis of tumors at different time points post i.v. injection of free ICG and ICG@HMOS@ZC. (“****” represents that the two groups of data are very different statistically) Reproduced with permission from Ref. [166]. Copyright 2022, Wiley Online Library.

Among the traditional chemotherapeutic agents, platinum (Pt)-based drugs, mainly including cisplatin (cis-diamine dichloroplatinum (CDDP)), oxaliplatin, and carboplatin, are one of the major clinically used anticancer drugs due to their potent cytotoxicity that disrupts DNA replication [164]. However, due to the drawbacks of rapid in vivo clearance, weak tolerance, and poor targeting of free CDDP molecules, clinical use often brings about side effects such as poor chemotherapeutic efficacy or even severe systemic toxicity [165]. Therefore, recent research is focused on finding efficient delivery vectors for CDDP with high loading rates, stability as well as targeting capability. Ma and co-workers have prepared sub-50 nm CDDP-loaded hollow mesoporous organosilica (HMOS) nanoparticles (termed as Pt@HMOS), which were subsequently decorated with the bimetallic Zn^2+^/Cu^2+^ co-doped MOF (termed as Pt@HMOS@ZC) to plug the pores of nanoparticles for efficiently preventing the premature leakage of CDDPs and improving the loading and delivery capacity of HMOS (Figure 7A) [166]. When Pt@HMOS@ZC entered the tumor cells, the acidic environment would cause the decomposition of outer MOF to release CDDP for the chemotherapy of cancer (Figure 7B). Simultaneously, free Cu^2+^ can be released in this process, which can deplete large amounts of reduced glutathione (GSH) in cancer cells and catalyze the decomposition of hydrogen peroxide (H_2_O_2_) into highly toxic ·OH in tumors via a Fenton-like reaction, which acted synergistically with CDDP for chemodynamic therapy of tumors (Figure 7C–E). The combination of bimetallic MOF and HMOS helps to create systems that intelligently unlock nanomedicines, a concept that offers new designs and ideas for the precise release of tumor drugs.

### 4.2. Bimetallic CPs Based on Cancer Vaccine

**Figure 8:**
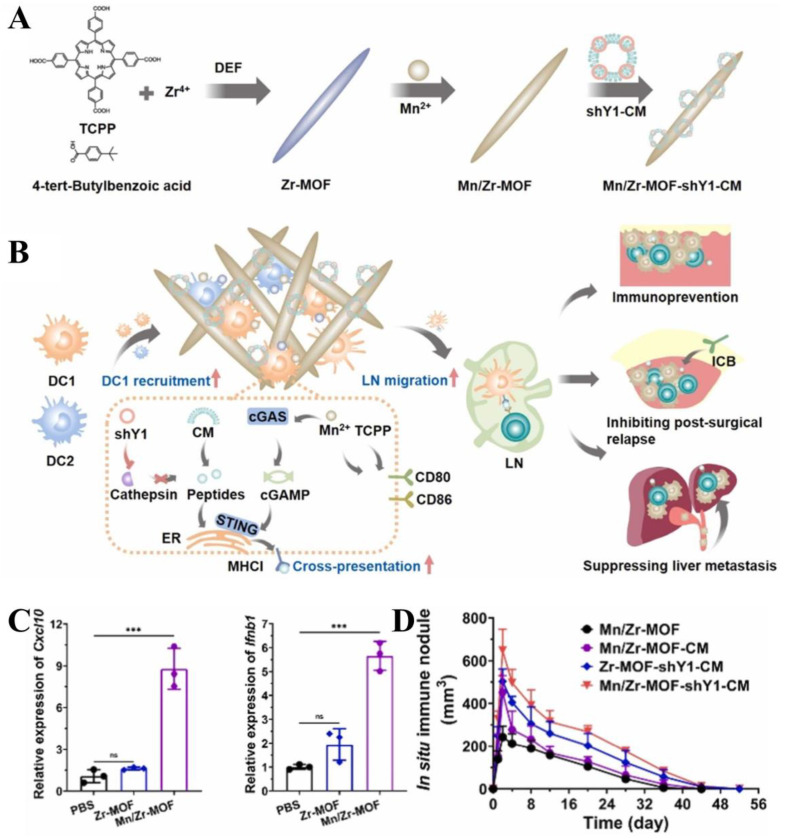
(**A**) Schematic illustration of the fabrication of Mn/Zr-MOF-shY1-CM. (**B**) Schematic representation of Mn/Zr-MOF-shY1-CM-induced immune response cascades. (**C**) QRT-PCR analysis of *Cxcl10* and *Ifnb1* expression in BMDCs. (“***” represents a statistically significant difference between the data, ns = not statistically) (**D**) In situ immune nodule measurement (n = 4). Reproduced with permission from Ref. [170]. Copyright 2024, Elsevier.

Cancer vaccines with easy mass production and a favorable safety profile are increasingly being explored for the immunological treatment of cancer [167]. In terms of immune mechanisms, tumor vaccines spatiotemporally coordinate antigen transport to lymph nodes (LNs), cytoplasmic delivery, and cross-presentation of antigen in dendritic cells (DCs) with innate immune stimuli to activate specific T cell responses [168]. However, limitations in the body’s own immune stimulation and DC spatiotemporal coordination (for example, DC recruitment, activation, and migration to LNs) severely affect their antitumor utility [169]. Therefore, there is an urgent need to develop an effective delivery system to solve the dilemma of cancer immunotherapy. Liu and co-workers developed a bimetallic organic framework, Mn/Zr-MOF, equipped with a biomimetic nanovaccine targeting Ythdf1 to create an in situ pro-inflammatory immune ecosystem for enhanced DC spatiotemporal coordination (Figure 8A) [170]. Plasmids expressing short hairpin RNA (shRNA) against Ythdf1 (shY1, downregulating Ythdf1 expression) and liposome-modified tumor cells membrane (CM) would be packaged and squeezed through a polymeric membrane in an extruder to form shY1-CM nanoparticles as a Ythdf1-targeted biomimetic nanovaccine. Mn/Zr-MOF-shY1-CM was fabricated by adsorbing shY1-CM nanoparticles onto Mn/Zr-MOF, which greatly enhanced the stability and targeting action of the vaccine (Figure 8B). The results from cellular and animal experiments revealed that the cancer vaccine exhibited a strong preventive effect in delaying B16-OVA and MC38 tumorigenesis (Figure 8C,D). Additionally, it demonstrated a robust therapeutic effect of inhibiting postoperative MC38 tumor recurrence and heterochronic liver metastasis. This well-designed bimetallic MOF-loaded cancer vaccine provides an efficient strategy for the fabrication of personalized scaffold cancer vaccines.

### 4.3. Bimetallic CPs for Chemodynamic Therapy (CDT)

**Figure 9:**
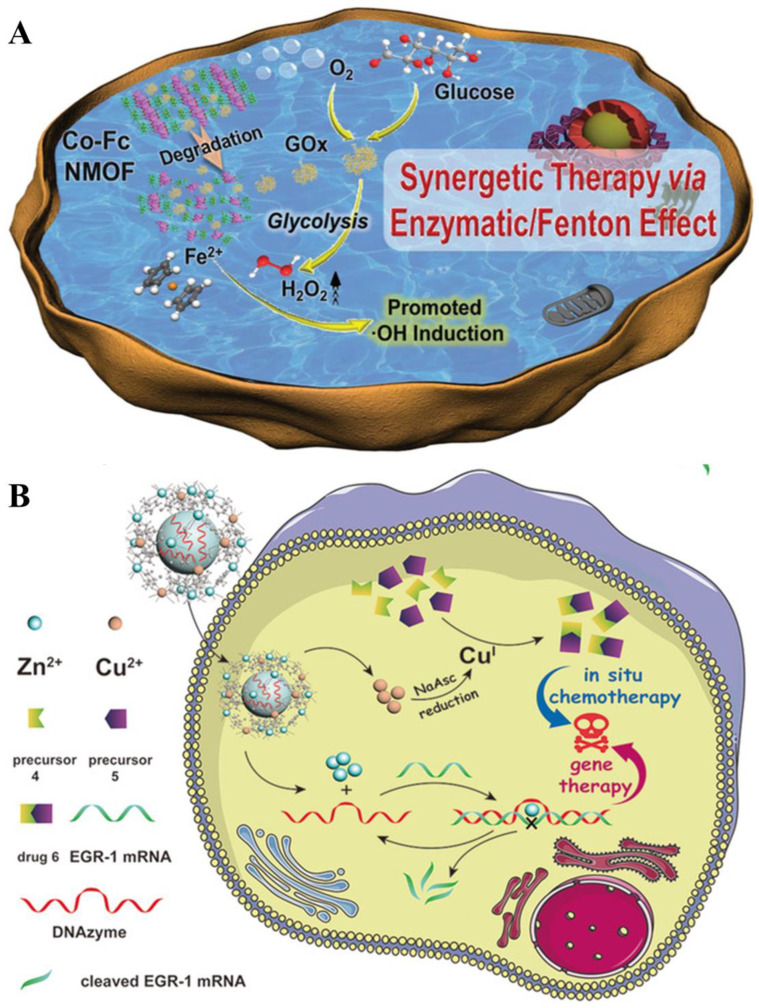
(**A**) Schematic illustration of Co-Fc@GOx as a cascade enzymatic/Fenton reaction platform for promoted •OH induction and enhanced therapeutic effects on cancer cells. Reproduced with permission from Ref. [174]. Copyright 2020, Wiley Online Library. (**B**) Schematic illustration of a smart DNAzyme@Cu/ZIF-8 nanoplatform for the synergistic chemo-gene therapy. Reproduced with permission from Ref. [175]. Copyright 2021, Wiley Online Library.

Chemodynamic therapy (CDT) has gained widespread attention for its use in cancer therapy by inducing reactive oxygen species (ROS) in tumor cells and disrupting the balance of the redox state in cancer cells [171]. CDT is driven by Fenton reactions which are based on the generation of highly toxic ROS from intracellular H_2_O_2_ catalyzed by Fe^2+^/Fe^3+^ redox pairs [172]. The resulting ROS has a strong oxidation capability and can cause severe oxidative damage to organelles and biomolecules. However, the Fenton reactions progress is overly dependent on the tumor microenvironment conditions such as pH and concentration of H_2_O_2_, hence the anticancer effect of CDT is greatly limited [173]. A novel cascade nanozyme (Co-Fc@GOx) combining nanoscale Co-ferrocene MOF and GOx was fabricated and showed remarkable cascade enzymatic/Fenton activity (Figure 9A) [174]. Owing to the synergistic effect of Fe^2+^ and Co^2+^, the prepared Co-Fc MOF can not only possess high Fenton activity but also bind more firmly to GOx. The results showed that the loaded GOx catalyzed a large amount of glucose in the tumor environment to produce abundant gluconic acid and H_2_O_2_, which significantly facilitated the Fenton reaction and accelerated the in situ induction of ROS, particularly ·OH, thereby enhancing the therapeutic effects on cancer cells. This Co-Fc@GOx can effectively regulate the tumor microenvironment through a cascade reaction and may serve as an alternative CDT platform to promote tumor therapy. Qu and co-workers have designed bimetallic CuZn-MOF (Cu/ZIF-8) wrapped with DNAzyme for intracellular in situ synthesis of tumor drugs and DNAzyme-based gene therapy (Figure 9B) [175]. The synthesized DNAzyme@Cu/ZIF-8 can release Cu^2+^, Zn^2+^, and DNAzyme upon decomposition in the acidic environment of tumor cells. The released Cu^2+^ underwent reduction to Cu^+^ through ascorbic acid, subsequently, these ions catalyzed the synthesis of chemotherapeutic drugs via the copper-catalyzed azide-alkyne cycloaddition (CuAAC) reaction. Moreover, the released Zn^2+^ can act as a cofactor to activate the cleavage activity of DNAzyme. Both the synthesis of anticancer drugs and the activation of gene therapy took place within the tumor cells, which could destroy the cancer cells in situ to minimize the side effects on normal organisms.

### 4.4. Bimetallic CPs for Radiotherapy

**Figure 10:**
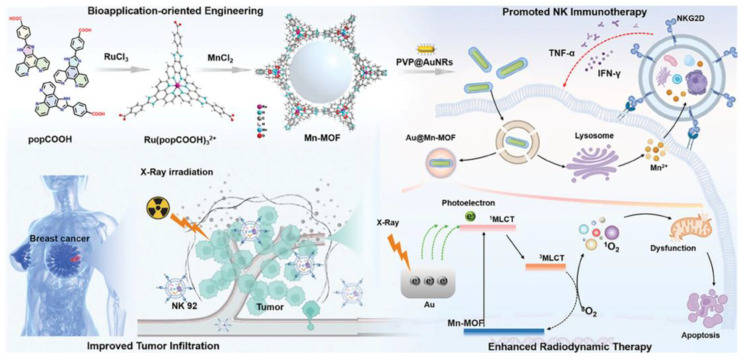
Schematic illustration for bio-functional Au@MnRu-MOF triggered enhancement of radiotherapy. Reproduced with permission from Ref. [181]. Copyright 2023, Wiley Online Library.

Radiotherapy is the most commonly used clinical treatment strategy for early and intermediate-stage tumors [176]. However, radiotherapy is often ineffective due to factors such as variations in the radiosensitivity of different types of tumor cells and infections in solid tumors [177]. Moreover, the potential resistance and side effects of radiotherapy (for example, esophagitis, enteritis, radiation cystitis, pulmonary fibrosis, bone marrow injury, and other side effects) seriously damage patients’ health [178]. Therefore, increasing the sensitivity of cancer cells to radiotherapy as well as reducing the impact of side effects of radiotherapy have become important directions in the development of radiotherapy. Gold nanoparticles as emerging radiosensitizers can both damage cells by generating free radicals through the photoelectric and Compton effects, as well as improve the efficiency of radiation therapy by inhibiting DNA repair processes [179]. The composite system of AuNPs and bimetallic MOFs achieved significant radiosensitization of tumors due to the synergistic effect of multiple components [180]. The Lu group designed and synthesized a bio-functional bimetallic MOF MnRu-MOF by doping Mn^2+^ into a ruthenium (Ru) complex, followed by the incorporation of gold nanorods (AuNR) into this system to prepare the heterojunction radiosensitizer Au@MnRu-MOF with enhanced cancer radionuclide/immunotherapy (Figure 10) [181]. Single-crystal XRD confirms that MnRu-MOF is a novel crystal structure with *P63/mmc* space group and a channel diameter of 27 Å. In this system, Au@MnRu-MOF can release Mn^2+^ under acidic conditions and modulate NK-mediated (NK = natural killer) cell therapy to overcome the proliferation of the triple-negative breast cancer cell line MDA-MB-231. Moreover, photoelectrons generated by high-energy X-ray excitation of AuNR can be transferred to the excited singlet state of Ru polypyridine complexes, promoting the accumulation of cytotoxic free radicals. As a result, the MnRu-MOF combined with AuNR was formed with a core-shell heterojunction structure and used to inhibit the proliferation of MDA-MB-231 cells, which provides ideas for the rational design of biologically functional MOFs and the combined treatment of cancer.

### 4.5. Bimetallic CPs for Immunotherapy

**Figure 11:**
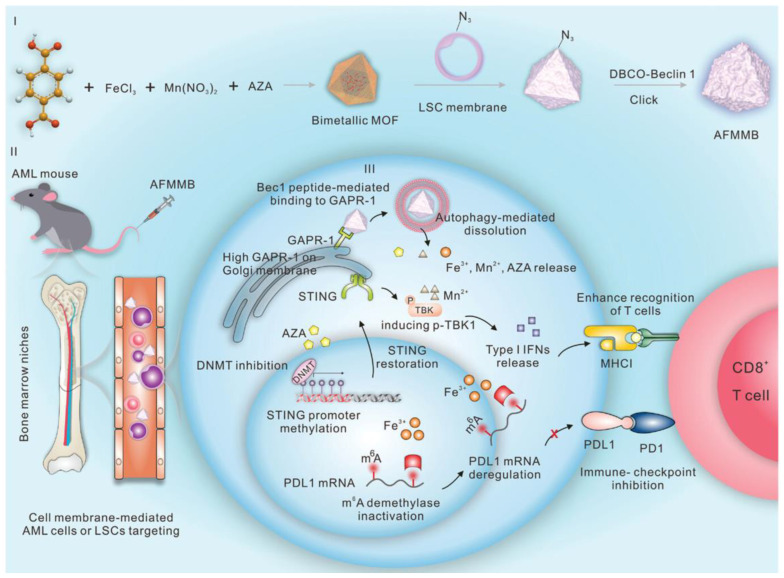
Schematic illustration of the successive fabrication of the AFMMB with DNA demethylation and RNA hypermethylation activities for enhancing antitumor immunity. Reproduced with permission from Ref. [184]. Copyright 2023, Wiley Online Library.

Immunotherapy is a novel anti-cancer tool that can recognize and kill cancer cells by activating the host’s own immune system [182]. However, acute myeloid leukemia (AML) and other malignant cells can activate a variety of immune evasion mechanisms to escape forced elimination by the autoimmune system. Among them, epigenetic alterations-mediated reduction in the antigenicity of leukemoblasts (LBs) is one of the key mechanisms of immune escape and resistance to T-cell immunotherapy [183]. Thus, the epigenome can be reprogrammed to reverse immune evasion, regarded as an emerging strategy for the treatment of multiple malignancies. Song and co-workers prepared a bimetallic MOF-based nanocomposite (called AFMMB) consisting of DNA hypomethylating agents azacitidine (AZA), leukemic stem cell (LSC) membranes, and pro-autophagic peptides for the immunological treatment of leukemia [184]. Due to the homing ability and immuno-compatibility of LSC membranes, the constructed AFMMB particles exclusively targeted LBs and triggered autophagy by binding to the Golgi-associated plant pathogenesis-related protein 1 (GAPR-1), leading to its disassembly and the release of Fe^3+^, Mn^2+^, and AZA (Figure 11). The release of DNA hypomethylating agents effectively suppressed DNA methylation, upregulated major histocompatibility complex class I molecules, and induced RNA methylation-mediated decay of programmed cell death protein ligand transcripts, thereby restoring stimulators of the interferon gene pathway. The dual epigenetic effects of AFMMB enhanced the antigenicity of AML cells and consequently facilitated the recognition and killing of cytotoxic T cells by tumor cells. This work highlighted the promising applications of bimetallic CPs for the treatment of hematological malignancies and solid tumors.

## 5. Conclusions and Outlook

Various bimetallic coordination polymers with superior properties have been developed. These bimetallic CPs are mainly constructed by the strategies of one-pot methods and post-synthesis modifications. One-pot synthesis is considered the most commonly employed method and is categorized into self-assembly and metal-ligand methods depending on the order of the second ions addition. For the post-synthesis approach, the bimetallic CPs are achieved by synthesizing monometallic CPs followed by the addition of a second ion including ion exchange and seed methods. Based on the systematic investigation and judicious design of the two metals and coordination with the organic ligands, bimetallic CPs with desirable properties and performance can be achieved. Multiple spectroscopic and morphological instruments and techniques are developed to investigate the composition, location, and arrangement of metals in synthesized bimetallic CPs.

Bimetallic CPs have the advantages of adjustable porosity, flexible luminescence, strong electrical conductivity, and high electrocatalytic activity. These materials can be widely used for biosensing and biomedical applications, such as the detection of metal ions and small molecules, immune response, as well as nucleic acids. In addition, it has been demonstrated that multifunctional bimetallic CPs combined with cancer drugs, cancer vaccines, or nanomaterials can be applied for efficient drug delivery and cancer therapy. In the drug delivery system, the bimetallic CPs not only act as a stable delivery platform due to the synergistic effect of the bimetals but also precisely release the drug, functional nanomaterials, or free ions in the tumor microenvironment. Moreover, the released metal ions can also form a composite superimposed therapeutic system with drugs and nanoparticles in the tumor cells to enhance the anticancer effect via chemodynamic therapy, radiotherapy, and immunotherapy.

The synergistic effect of multiple metal ions in bimetallic CPs is the main reason for their enhanced properties, but it is highly dependent on the uniform distribution of both metals in the structure. The inhomogeneous distribution of the two metal ions would cause a significant decrease in sensing performance and transport stability as well as poor cancer therapy. Thus, more advanced instruments and techniques need to be developed to determine whether the two metal elements are uniformly distributed in the structure to guide the controlled synthesis of bimetallic CPs. Moreover, it is significant to develop strategies to precisely control the incorporation of metals and construct the bimetallic CPs at the atomic level. It is anticipated that many novel functional bimetallic CPs can be designed for wide potential applications.
